# Burnout, resilience, and subjective well-being among Portuguese lecturers’ during the COVID-19 pandemic

**DOI:** 10.3389/fpsyg.2023.1271004

**Published:** 2023-11-30

**Authors:** Luísa Castro, Carla Serrão, Ana Rita Rodrigues, Sílvia Marina, José Paulo Marques dos Santos, Teresa Sofia Amorim-Lopes, Carla Miguel, Andreia Teixeira, Ivone Duarte

**Affiliations:** ^1^Department of Community Medicine, Information and Decision in Health; Faculty of Medicine, University of Porto, Porto, Portugal; ^2^CINTESIS@RISE, MEDCIDS, Faculty of Medicine of the University of Porto, Porto, Portugal; ^3^Escola Superior de Educação do Instituto Politécnico do Porto, Porto, Portugal; ^4^Centre for Research and Innovation in Education (inED), Porto, Portugal; ^5^University of Maia, Maia, Portugal; ^6^Center for Psychology, University of Porto, Porto, Portugal; ^7^Department of Biomedicine, Faculty of Medicine, University of Porto, Porto, Portugal; ^8^Faculty of Medicine, University of Porto, Porto, Portugal; ^9^ADiT-LAB, InstitutoPolitécnico de Viana do Castelo, Rua Escola Industrial e Comercial Nun’Álvares, Viana do Castelo, Portugal

**Keywords:** burnout, COVID-19, lecturers, resilience, subjective well-being

## Abstract

**Background:**

Lecturers face a large wide of occupational stressors. If the prolonged stress and the symptomatology associated with the working conditions to which lecturers were exposed were already a concern before the pandemic, the pandemic may have exacerbated this psychosocial vulnerability. Burnout is a psychological syndrome that develops in response to chronic work stress. This study aims to describe burnout amongst lecturers working in Portugal and to analyse potential determinants of burnout during the COVID-19 pandemic.

**Methods:**

A cross-sectional study was performed using an online questionnaire distributed via social networks. The survey collected sociodemographic and sleep patterns data in addition to applying the Copenhagen Burnout Inventory (personal, work- and student-related burnout), the Resilience Scale, the Depression Anxiety and Stress Scales, and the Satisfaction with Life Scale.

**Results:**

The sample included 331 lecturers from 35 different colleges and faculties. Three significant models explained personal (R2 = 54%), work- (R2 = 47%) and student- (R2 = 19%) related burnout. Lower levels of resilience and higher levels of depression and stress were significantly associated with personal and work-related burnout. Changes in sleep patterns were additionally associated with both personal and work-related burnout.

**Conclusion:**

Higher education institutions must recognize the impact of the work environment and organizational culture on faculty mental health and take proactive measures to improve this environment. These institutions can implement support strategies such as educational technology training, professional development programmes, emotional support resources, and workload flexibility. Implementing measures to enhance lecturers’ resilience and overall life satisfaction could potentially help mitigate burnout and improve the well-being of educators, ultimately contributing to the overall quality of education.

## 1 Introduction

The COVID-19 pandemic has affected all fields in society. Although the pandemic poses a universal hazard to all occupational categories, it is possible that the number of stressors in certain classes is substantially higher and more challenging, as is the case of the teaching profession. In most countries around the world, from kindergarten to the doctoral level, there have been closures of schools and higher education institutions and cessation of face-to-face education. In fact, the COVID-19 pandemic “generated the largest disturbance of education systems in history” (De Giusti, 2020). This unprecedented situation has given rise to the development of “emergency remote teaching” (Hodges et al., 2020). Teachers did not have enough time to adequately prepare for the challenges presented by the abrupt transition to teleworking; in a relatively short period of time, the whole of the academic community began teaching in front of a computer screen, without essential training, resources, and digital skills to face these demands (De Giusti, 2020; Gatti et al., 2020; Hodges et al., 2020).

In Portugal, like in many countries, prior to COVID-19 lockdown, “the vast majority of courses currently on offer were pedagogically designed to be delivered face-to-face” (Gatti et al., 2020). Between March and May, the instruction was 100% virtual. In the final of May 2020, and with the easing of restriction measures, some Portuguese higher education institutions have decided to keep classes running online only. In other cases, lecturers had to adapt to new measures, namely hybrid education (some students attend the class in person, whilst others join virtually remotely). New work situations have arisen due to the need to implement very restrictive protocols to ensure that the virus does not spread: ensuring physical distance, ensuring the cleanliness and disinfection of the classrooms, responding to situations where students appeared ill, etc. In addition, teachers and students had to deal with the fear of possible infection. This atypical scenario created significant challenges, which may have caused multiple problems such as stress, depressive symptoms and sleep problems (Rana et al., 2020; Restubog et al., 2020). This new situation may have increased the number of work-related tasks previously identified in academic field research. According to previous research, work overload, the homework interface, and the need to increase scientific productivity are the main factors contributing to lecturers’ stress (Gomes et al., 2013). A variety of stressful situations that can trigger emotional exhaustion and reduced job satisfaction have been identified by a review of burnout in lecturers: work pressure, conflicts at work, large classes, learning difficulties, and students’ behaviours (Fiorilli et al., 2015). In addition to these usual stressors, the pandemic has created new demands for teachers. Lectures faced a set of challenges, particularly, in preparation of the materials, in developing and delivering the contents, performing the lesson, following students’ growth, and integrating other software’s into one learning platform. Alongside increased work intensity, the pandemic is a stressful event that involves uncertainty and unpredictability (Gatti et al., 2020; Hodges et al., 2020). This scenario, when not handled or alleviated, may lead to burnout (Vazi et al., 2013) a “state of physical, emotional and mental exhaustion that results from long-term involvement in work situations that are emotionally demanding” (Schaufeli and Greenglass, 2001).

Teachers had to suddenly transition to digital education. This entailed the forced acquisition of a new teaching method, which, overall, became a source of stress for a significant portion of educators. This situation amongst education professionals carries consequences for students. Students with exhausted teachers tend to exhibit reduced motivation for schoolwork. Furthermore, other research indicates that the psychological state of teachers impacts the academic outcomes of children and adolescents, often resulting in lower academic achievements and greater difficulties in achieving success in school. Poor working conditions were found to be predictors of emotional exhaustion and low personal accomplishment (Fernández-Martínez et al., 2017).

Reports on burnout levels amongst higher education teachers seem to be similar to those reported amongst health-care workers (Watts and Robertson, 2011). In a systematic review performed by Khan (2019) assessing the origins and adverse consequences of burnout, it was found that university academicians were exposed to personal and environmental demands (Khan, 2019). From family relationships, expectations, attitudes, beliefs and perceptions, personality, and personal lifestyle habits (such as sleeping habits), to environmental demands related to work, professional roles, time pressures, relationships and lack of resources, these professionals appear to be subject to several demands. Nevertheless, psychological resilience (Wagnild and Young, 1993; Serrão et al., 2021) and satisfaction with life have been revealed as protective variables, and hence not all individuals exposed to the aforementioned demands, develop stress, anxiety, depression or burnout (Wagnild and Young, 1993; Melillo and Suarez Ojeda, 2005). According to a recent cross-sectional quantitative study on 831 lecturers and professors of a Mexican public university, cynicism and emotional exhaustion exhibited the strongest correlation with accomplishment in teaching, right after resources availability (García-Rivera et al., 2022). Psychological resilience refers to the human ability to cope with high-risk circumstances and regain socio-emotional balance, whilst resilience fosters individual regulation moderating the adverse effects of stress (Wagnild and Young, 1993; Serrão et al., 2021). Subjective well-being, like psychological resilience, derives from positive psychology. Two components underly well-being: an emotional component (hedonic well-being; subjective emotions such as the experience of pleasure and happiness) and a cognitive component (eudaimonic well-being; ones’ motivation to achieve goals that promotes positive feelings) (Diener et al., 1985; Ryff, 1989; Culbertson et al., 2010). This last category is termed “life satisfaction” and relates to the rational judgement and evaluation that individuals make about the quality of their lives, including expectations, comparisons to others, and other cultural traits (Diener et al., 1985).

As mental resilience facilitates the process of coping with highly stressful situations (Campbell-Sills and Stein, 2007), it can potentially mitigate the symptoms of burnout. These symptoms may include fatigue, difficulty in managing one’s emotions, and a sense of detachment or aloofness in interpersonal relationships. Conversely, mental resilience can bolster feelings of professional achievement and the ability to handle job demands (Bakker and Demerouti, 2007).

Prior research has indicated that women are more susceptible to challenges related to emotional well-being during periods of confinement. This vulnerability is partly attributed to their pre-existing lower levels of mental health (Lacomba-Trejo et al., 2022). Additionally, women often face the added burden of balancing their professional responsibilities with family duties, including caring for children and dependents (Tugend, 2020). Conversely, shorter sleep duration and lower sleep quality are associated with increases in the levels of perceived stress (Kim et al., 2019; Alotaibi et al., 2020). These relationships have also been evidenced during the COVID-19 pandemic.

According to a study of 103 educators in South Africa, as levels of disengagement and exhaustion decreased, levels of subjective well-being increased, thus suggesting that subjective well-being may preclude experiences of exhaustion and disengagement amongst educators (Hansen et al., 2015).

However, little research has been conducted in higher education compared to other professions (Watts and Robertson, 2011). Besides, another critical matter is the scarce attention paid to identify protective variables against burnout. Furthermore, solutions to mitigate burnout amongst lecturers should explore factors associated with positive psychology and well-being, which can be used as personal resources against this phenomenon. By developing their personal resources, academicians can develop adaptive strategies to minimise the adverse effects of work demands (Ghorpade et al., 2011). Psychological resilience and satisfaction with life, which are reported to potentially aid during times of distress (Vazi et al., 2013), may protect workers from burnout and chronic stress. Identifying protective variables against burnout is important when designing health promotion programmes for lecturers. For example, a recent study has attained satisfying results in a sample of 80 Nigerian university history lecturers with high burnout levels, using online psychological interventions targeting job burnout reduction (Eseadi et al., 2023) Moreover, different disruptions are expected in the future, namely new waves and new diseases affecting the whole world, it is important to study whether there are variables positively associated with teacher achievement in these challenge times. The ability to be resilient has proven indispensable during confinement. More resilient individuals have reported enhanced subjective well-being and life satisfaction, as indicated by Macintyre et al. (2020). Additionally, they have exhibited higher levels of emotional functioning (Lacomba-Trejo et al., 2022).

A better understanding of the potential risk for burnout outcomes amongst lectures is crucial for comprehending the impact of COVID-19 during the period of confinement. The aim of this study is to describe burnout amongst lecturers working in Portugal and to analyse potential determinants of this burnout during the COVID-19 pandemic and to investigate how concerns related to COVID-19, emotional balance, life satisfaction, and resilience may mediate the emotional impact. Burnout amongst lectures is very important to discuss, as it has profound implications for the quality of education, the mental health of teachers and the general well-being of society. It is essential to understand and address these issues to promote a healthy and effective academic environment, especially during times of significant change such as the pandemic.

## 2 Method

### 2.1 Study design

This is a cross-sectional, quantitative study utilising a web-based survey applied to lecturers working in public or private Portuguese higher education institutions. The survey was implemented using the Google Forms® platform and was available during the period of declared national calamity (between 19 June and 31 July, 2020). This study was conducted in line with the Declaration of Helsinki and received approval from the Ethics Committee of the São João Hospital Center (98/2020, on 29 May, 2020). All participants gave informed consent online in compliance with the General Data Protection Regulation guidelines for clinical research (Marina et al., 2020).

The survey was shared via social networks and promoted by university and college institutions.

### 2.2 Participants

This study consisted of lecturers who were working in Portugal when the COVID-19 pandemic started. No other eligibility criteria existed.

A total of 331 lecturers (M ± SD age 48.2 ± 9.7 years old, 65.9% females) from across the country participated in this study. Nearly 67% (*n* = 223) had over 15 years of teaching experience and 86.1% (n = 285) worked in public higher education institutions, whilst 6 worked in public and private higher education institutions. In this sample, 88.2% (*n* = 292) were teleworking and 11.8% (*n* = 39) were working in-person. Almost all participants (*n* = 322; 97.3%) used online instruction. Regarding academic qualifications, 226 (68.3%) had a post-doctoral, aggregation or doctoral degree, 75 (22.7%) had a master’s degree and 30 (9.1%) had a bachelor’s or graduate’ degree. Of the 241 (72.8%) participants who had children, 44.4% had children aged 12 years or younger. A total of 63 (19%) participants were caregivers for older people or people with disabilities. More than half of the participants (59.4%) noticed a change in their sleep pattern during the pandemic. The most commonly reported sleep symptoms were disrupted sleep (75.5%) and sleep latency (time taken to fall asleep) (63.8%).The characteristics of the participants are summarised in Table 1. Given the sample size of 331 participants, for a confidence level of 95%, and assuming the most conservative scenario of 50% estimated proportion, a margin of error of 5.39% is estimated for the proportions computed.

**Table 1:** Characteristics of sample participants (*n* = 331).

Gender*, *n* (%)	
Male	112 (33.9)
Female	218 (66.1)
Marital status, *n* (%)	
Single	60 (18.1)
Married/nonmarital partnership	230 (69.5)
Divorced or separated	39 (11.8)
Widowed	2 (0.6)
Sleep latency, *n* (%)	
Never	120 (36.3)
Sometimes	182 (55.0)
Most of the time	29 (8.8)
Disrupted sleep, *n* (%)	
Never	81 (24.5)
Sometimes	184 (55.6)
Most of the time	66 (19.9)
Hypersomnia, *n* (%)	
Never	263 (79.5)
Sometimes	66 (19.9)
Most of the time	2 (0.6)
Sleep hours, *n* (%)	
< 6 h	66 (19.9)
6 h - 8 h	252 (76.1)
> 8 h	13 (3.9)
Change in sleep routines*, *n* (%)	
None	134 (40.6)
Sleep less than usual	99 (30.0)
Sleep more than usual	18 (5.5)
Went to bed later than usual and woke up later than usual	65 (19.7)
Went to bed earlier than usual and woke up earlier than usual	14 (4.2)
Professional experience, *n* (%)	
Five years or less	37 (11.2)
Between 6 and 15 years	71 (21.5)
More than 15 years	223 (67.4)
Type of higher education institutions, *n* (%)	
Public	285 (86.1)
Private	40 (12.1)
Both	6 (1.8)
Online instruction, *n* (%)	
Yes	322 (97.3)
No	3 (0.9)
Other	6 (1.8)
Previous experience in remote teaching, *n* (%)	
Yes	73 (22.1)
No	258 (77.9)

*1 participant opted not to answer to the question.

### 2.3 Measures and covariates

Sociodemographic data were collected using a self-administered questionnaire. Psychological variables were collected using the Copenhagen Burnout Inventory (CBI); the Resilience Scale; the Depression, Anxiety, and Stress Scales (DASS-21); and the Satisfaction with Life Scale (SWLS).

Burnout was measured by the validated Portuguese version of the CBI (Kristensen et al., 2005; da Fonte, 2011). The CBI is a 19-item tool with three subscales: personal, work-related, and student-related burnout. The personal burnout subscale measures feelings of physical, emotional, and mental fatigue and exhaustion. The work-related burnout subscale assesses the symptoms that respondents’ attribute to work. The student-related burnout subscale describes feelings of physical and psychological fatigue and exhaustion that respondents attribute to their work with students. All items are scored on a 5-point Likert scale. The score for each subscale is the average of item scores within the subscale and ranges from 0 to 100. Scores ≥50 in each of the three subscales were considered high-level burnout (Kristensen et al., 2005; da Fonte, 2011). These subscales are characterised by high internal consistency. In this study, the Cronbach’s alphas (α) were 0.93, 0.88, and 0.85 for personal burnout, work-related burnout, and student-related burnout, respectively.

The Resilience Scale (Wagnild and Young, 1993; Oliveira and Machado, 2011) includes 25 items scored on a 7-point Likert scale ranging from strongly disagree (one) to strongly agree (seven). The total score is determined by the sum of the 25 items, ranging from 25 (low psychological resilience) to 175 (high psychological resilience). The Portuguese version presented high internal consistency, α = 0.89 (Oliveira and Machado, 2011). In this study, α = 0.93.

DASS-21 (Lovibond and Lovibond, 1995; Ribeiro et al., 2004) was used to evaluate mental health symptoms. This version consists of a 21-item, 4-point Likert questionnaire that includes three self-reported subscales designed to measure the negative emotional states of depression, anxiety, and stress. Each of the three subscales contains seven items scored on a scale of zero (did not apply to me at all) to three (applied to me very much or most of the time). For each subscale, a total score is calculated by the sum of the seven items and is then classified in the following categories: normal (depression: from 0 to 4; anxiety: from 0 to 3; stress: from 0 to 7); mild (depression: from 5 to 6; anxiety: 4; stress: from 8 to 9); moderate (depression: from 7 to 10; anxiety: from 5 to 7; stress: from 10 to 12); severe (depression: from 11 to 13; anxiety: from 8 to 9; stress: from 13 to 16) and extremely severe (depression: from 14 to 21; anxiety: from 10 to 21; stress: from 17 to 21). In the current study, α = 0.88, 0.86, and 0.90 for the depression, anxiety, and stress subscales, respectively.

SWLS (Diener et al., 1985; Simões, 1992) is a 5-item 5-point Likert scale that assesses an individual’s global judgement regarding life satisfaction. The versions of this scale have acceptable or high internal consistency; in the original version, α = 0.87 (Diener et al., 1985) and in the Portuguese version, α = 0.77 (Simões, 1992). In this study, α = 0.85. In the Portuguese version, the scale has no cut-off point and has a possible range from 5 (lower satisfaction with life), to 25 (higher satisfaction with life) points.

### 2.4 Data analysis

Data from Google Forms® were exported in a Microsoft Excel® 2016 spreadsheet, USA, and all statistical analyses were carried out using SPSS® Statistics (version 26.0; SPSS Inc., Chicago, Illinois, United States) and Jamovi 1.1.9.0. Absolute and relative frequencies, *n* (%), were used to describe categorical variables. Normally distributed quantitative variables were described by means and standard deviations (M ± SD). Non-normally distributed quantitative variables were described by medians (Med) and interquartile intervals [Q1; Q3]. Normality was verified by observing the histograms.

Pearson’s or Spearman’s coefficient was also used to explore the association between different domains (resilience, anxiety, depression, stress, life satisfaction and burnout). The internal consistency of each of the subscales was assessed using Cronbach’s alpha (α) and a value above 0.7 was considered acceptable (Kline, 2023).

A separate multiple linear regression analysis was performed for each outcome (personal, work-related, and student-related burnout). The independent variables included in each multiple regression were chosen by performing simple linear regressions with each variable in the dataset identified as a potential predictor of burnout, including socio-demographics variables, variables related to COVID-19 and variables obtained from questionnaires (resilience, SLWS, and subscales from DASS-21). All variables that correlated with the outcomes at *p* ≤ 0.20 in the simple regression were included in the multiple linear regression analyses. Only the significant variables were maintained in the final multivariate models for personal, work-related, and student-related burnout.

The results of the linear regressions are presented with unstandardised coefficient values (β), their respective 95% confidence intervals (95% CIs) and *p*-values. The final model was evaluated using the F statistic of the overall model test, p-values, and coefficients of determination (R2). The assumptions of the linear regression models were verified as follows: (a) visual analysis of the histogram to assess the normality of residuals; (b) a t-test to determine whether mean residuals were equal to zero; and (c) plots of residuals versus the fitted predictive values to check for homoscedasticity. Values of *p* ≤ 0.05 were considered significant.

## 3 Results

### 3.1 Results of levels of burnout dimensions and psychological variables

In this study, the levels of burnout amongst lecturers were assessed and categorized into two groups: low and high burnout. Notably, a substantial proportion of lecturers reported experiencing high levels of burnout in different domains. Specifically, 164 lecturers (49.5%) reported experiencing high levels of personal burnout. Additionally, 121 lecturers (36.6%) reported high levels of work-related burnout, highlighting the challenges associated with their professional roles. Moreover, 36 lecturers (10.9%) reported high levels of student-related burnout.

In terms of resilience, the majority of lecturers demonstrated varying degrees of resilience. Specifically, 162 lecturers (48.9%) exhibited moderate levels of resilience, 123 lecturers (37.2%) displayed high resilience, and 46 lecturers (13.9%) reported reduced resilience. Anxiety (77.3%), depression (75.8%) and stress (70.7%) were at normal levels amongst most participants (Table 2). The lecturers also demonstrated high satisfaction with life, with a median of 19 [17; 21] points.

**Table 2:** Descriptive statistics for burnout dimensions, resilience, anxiety, depression, and stress, of the 331 participants.

Variable	*n* (%)
Personal burnout	
Low	167 (50.5)
High	164 (49.5)
Work-related burnout	
Low	210 (63.4)
High	121 (36.6)
Student-related burnout	
Low	295 (89.1)
High	36 (10.9)
Resilience	
Low	46 (13.9)
Moderate	162 (48.9)
High	123 (37.2)
Anxiety	
Normal	256 (77.3)
Mild	19 (5.7)
Moderate	28 (8.5)
Severe	14 (4.2)
Extremely severe	14 (4.2)
Depression	
Normal	251 (75.8)
Mild	32 (9.7)
Moderate	29 (8.8)
Severe	12 (3.6)
Extremely severe	7 (2.1)
Stress	
Normal	234 (70.7)
Mild	33 (10.0)
Moderate	41 (12.4)
Severe	15 (4.5)
Extremely severe	8 (2.4)

Burnout was positively associated with low psychological resilience, low satisfaction with life, high depression, anxiety and stress. The associations between burnout and all these measures were all statistically significant (*p* < 0.001) (see Table 3).

**Table 3:** Associations between burnout dimensions, depression, anxiety, stress, resilience and satisfaction with life measures.

	Work-related burnout	Student-related burnout	DASS Depression	DASS Anxiety	DASS Stress	Resilience	Satisfaction with life
Personal-related burnout	0.778*	0.264*	0.523*	0.514*	0.622*	−0.386*	−0.275*
Work-related burnout	–	0.398*	0.575*	0.534*	0.632*	−0.432*	−0.356*
Student-related burnout	–	–	0.308*	0.262*	0.278*	−0.330*	−0.346*
DASS Depression	–	–	–	0.636*	0.659*	−0.521*	−0.503*
DASS Anxiety	–	–	–	–	0.665*	−0.341*	−0.299*
DASS Stress	–	–	–	–	–	−0.375*	−0.316*
Resilience	–	–	–	–	–	–	0.402*

*p < 0.001. DASS, Depression Anxiety and Stress Scales.

### 3.2 Results of personal burnout, work-related burnout, and student-related burnout subscales: multivariate analysis

Three significant multivariate models explained personal (R2 = 54%; *p* < 0.001), work- (R2 = 47%; p < 0.001) and student-related burnout (R2 = 19%; *p* < 0.001) (Table 4). Lower levels of subjective well-being and psychological resilience were significantly associated with work-related burnout (β = −0.51 and β = −20, respectively, where β is the unstandardised regression coefficient), and student-related burnout (β = −1.35, β = −0.21). Higher levels of depression and stress were significantly associated with personal (β =1.17, β =1.57, respectively) and work-related burnout (β =0.86, β =1.56).

**Table 4:** Unstandardised regression coefficients for CBI subscales as outcomes and socio-demographic, professional, and emotional variables as predictors from multiple linear regressions.

Variables	Personal burnout	Work-related burnout	Student-related burnout
	β [95% CI]	β [95% CI]	β [95% CI]
Gender			
Male	Reference	Reference	
Female	6.16 [2.46; 9.86]***	4.53 [1.19; 7.86]**	
Parental status			
Yes, aged 12 years or older	Reference		
No	4.26 [−0.06; 8.58]		
Yes, aged 12 years or younger	4.56 [0.49; 8.61]*		
Disruptive sleep			
Never			Reference
Sometimes			4.83 [0.22; 9.45]*
Most of the time			4.66 [−1.20; 10.52]
Sleep latency			
Never	Reference		
Sometimes	4.40 [0.49; 8.32]*		
Most of the times	5.44 [−1.81; 12.68]		
Changes in sleep patterns			
No	Reference	Reference	
Yes	9.07 [5.31; 12.82]***	5.04 [1.74; 8.34]**	
Sleep duration			
Less than 6 h	Reference		
6 h to 8 h	−3.81[−8.29; 0.67]		
More than 8 h	−19.09 [−28.92; −9.25]***		
Current work regime			
Present at the workplace	Reference		
Teleworking	6.24 [2.38; 10.10]**		
Resilience	−0.15 [−0.27; −0.04]**	−0.20 [−0.30; −0.09]***	−0.21 [−0.33; −0.10]***
Satisfaction with life		−0.51 [−1.02; −0.01]*	−1.35 [−1.90; −0.80]***
Depression	1.17 [0.47; 1.87]***	0.86 [0.19; 1.52]*	
Stress	1.57 [1.02; 2.11]***	1.56 [1.06; 2.06]***	
*R^2^*	0.544	0.470	0.190
*F*	31.4***	47.7***	19.1 ***

CI, confidence interval. *p ≤ 0.05; **p ≤ 0.01; ***p ≤ 0.001.

Additionally, being female (β =6.16, β =4.53) and changes in sleep patterns (β =9.07, β =5.09) were both associated with higher levels of personal and work-related burnout. Sleep duration was associated with personal burnout. Lecturers who slept more than 8 h had an average of 19.09 fewer points in personal burnout compared to lecturers who slept less than 6 h.

Having children aged 12 years or younger was associated with higher levels of personal burnout (β = 4.56) in comparison with having older children.

## 4 Discussion

COVID-19 has severely disrupted teaching, learning, research and mobility. The pandemic has brought huge challenges to the academic community (Gatti et al., 2020). This global scenario has given rise to the development of emergency remote teaching (Hodges et al., 2020) as a strategy to avoid the spread of the virus. Emergency remote teaching (Hodges et al., 2020) has added to the stresses and workloads experienced by higher education teachers who were already under pressure to balance teaching, research and service obligations whilst maintaining a work-life balance (Houston et al., 2006; Houlden and Veletsianos, 2020). Therefore, the main objective of this study was to describe burnout amongst lecturers and to analyse potential determinants of this phenomenon during the COVID-19 pandemic. To the best of our knowledge, this study is one of the first to assess the burnout faced by lecturers working in Portugal throughout the pandemic.

The results of this study indicate that 49.5% of lecturers experienced high levels of personal burnout, 36.6% experienced work-related burnout and 10.9% experienced student-related burnout. The prevalence of burnout observed in this study was higher than in previous studies conducted in Portugal, finding that 6.3 to 34.8% of lecturers could have symptoms characteristic of burnout (Houlden and Veletsianos, 2020). In a study by Oliveira (2011), 14.8% of respondents showed signs of emotional exhaustion, 9% had signs and symptoms of lack of professional fulfilment and 1.1% exhibited depersonalisation.

These data are not surprising, given that this professional field is considered to be very demanding (Vazi et al., 2013; Fiorilli et al., 2015). In recent years, national and international competitiveness in academia has been increasing. Teachers must juggle organisational activities, research activities, scientific productivity and pressure to publish with pedagogical activities (Gomes et al., 2013). Compounding this, the new context of emergency remote teaching has implied an ongoing adaptation process and created other sources of stress, related with teaching and learning remotely.

Our results highlight the multidimensionality of burnout syndrome. Indeed, each of the three burnout dimensions was linked with a particular set of covariates.

We found that gender significantly contributed to the outcomes. Our findings suggest that female gender is associated with higher levels of personal burnout and work-related burnout, which is in line with previous research developed in European or American contexts (Tümkaya, 2007; Ghorpade et al., 2011; Redondo-Flórez et al., 2020). Similarly Oliveira (2011) and Mota-Cardoso et al. (2002) concluded that Portuguese female university teachers had significantly higher values of emotional exhaustion when compared to men. Some studies point out that the difficulties experienced in reconciling multiple roles continue to be more constant and intense for women. In fact, a recent study conducted during October 2020 of 1,122 American teachers (Tugend, 2020), found that 74% of women feel their work-life balance has deteriorated compared to 63% of men and 82% of women teachers said their workload has increased compared to 70% of men. Indeed, whilst work-related responsibilities seem to be shared by men and women, the same does not happen in the family context, given that domestic demands and responsibilities (housekeeping and caregiver responsibilities) continue to fall primarily upon women (Matias et al., 2011). This reality lays some obstacles to a good balance between the two domains of life for both genders, since the choice between one domain in the other may generate negative consequences for the individuals’ health and well-being, namely the development of psychosocial risks, such as burnout, and decreased job satisfaction. In the Portuguese context, this gender inequality in the sharing of domestic responsibilities is changing, to the extent that men are gradually beginning to more actively participate in this role that was once exclusively attributed to women (Goncalves et al., 2018). Although a change in attitudes is beginning to occur, the female gender continues to assume greater responsibility and concern domestic matters, resulting in difficulty reconciling multiple roles (i.e., mother, wife, daughter, caregiver and housewife) (Watts, 2009).

Our results show that lecturers with children aged 12 and under were more likely to experience personal burnout than lectures without children or children aged 12 or older. In the same line of research, another study (Duarte et al., 2020) found similar results amongst healthcare workers. In addition to the fact that teachers work at a distance, their children are also being taught in the same format, implying additional work for parents. Non-school aged children, meanwhile require other forms of care from their parents.

The lockdown in response to COVID-19 resulted in lifestyle changes that affected sleep quality (Romero-Blanco et al., 2020). Our findings show that changes in sleep patterns are associated with higher levels of personal and work-related burnout and sleep duration was associated with personal burnout. Lecturers who slept more than 8 h had lower levels of personal burnout compared to those who slept less than 6 h. Sleep is restorative for daily functioning, and both the quality and quantity of sleep affect the individual’s ability to cope with emotional events (Vandekerckhove and Cluydts, 2010). In turn, sleep deprivation triggers negative emotional responsiveness and decreases the effect of positive emotions (Vandekerckhove and Cluydts, 2010). Practises and behaviours, such as sleep hygiene, are important for promoting consistent and uninterrupted sleep (Suni, 2022). Previous research has found that the use of a sunrise alarm clock in combination with the removal of electronic devices at bedtime was effective in improving sleep quality and reducing burnout scores (Brubaker et al., 2020). Another study (Gupta et al., 2020) showed that the COVID-19 lockdown was linked to changes in sleep timing and in the quantity and quality of night sleep.

Additionally, the risk variables associated with personal and work-related burnout are depression and stress symptoms. Although burnout and stress are different, they are closely related and generally linked to identical work-based psychosocial factors (Pines and Keinan, 2005; Vazi et al., 2013). Stress was defined as a tendency to overreact to a stressful event, with symptoms of tension and irritability, and burnout was defined as the psychological strain that results from exposure to stressful situations (Maslach and Jackson, 1981; Ghorpade et al., 2011).

The need to adapt to an unprecedented teaching model for the majority (in our sample, 77.9% had no previous experience of distance learning), technical problems with the use of online platforms, difficulties in social interaction, difficulty in following students and, in effect, professional life began to count all the hours of the day, making it not easy to separate professional life and family life. The use of teaching platforms by teachers was a source of concern as a very large percentage had never been used. Therefore, training may be the most appropriate solution, as this new learning environment requires adaptation.

As suggested in the literature on occupational burnout, personal characteristics can influence burnout levels and buffer negative aspects of the work environment (Sabagh et al., 2018). This study shows that personal characteristics such as resilience and life satisfaction (subjective well-being) predicted lower levels of work-related and student-related burnout.

Psychological resilience and life satisfaction were shown to be protective variables against burnout. In this sense, higher education institutions must develop and provide health programmes to promote and sustain resilience amongst lecturers. Such health programmes, like mindfulness-based interventions (MBI) may improve these protective factors, and safeguard teachers against occupational stress and burnout (Lomas et al., 2017). Previous studies (Paquette and Rieg, 2016; Hwang et al., 2017) report that these psychoeducational programmes are effective in reducing levels of burnout, anxiety, stress, depression and also in promoting teachers’ well-being. Thus, including MBI in teacher education must be considered in order to help maintain good psychological health amongst teachers (Jennings et al., 2017; Ruijgrok-Lupton et al., 2018). In addition, this type of mental training also seems to be an asset for teachers in the performance of their duties, as it promotes adaptive emotional regulation, a sense of self-efficacy and subjective well-being (Jennings and Greenberg, 2009; Emerson et al., 2017).

### 4.1 Limitations and strengths

Some limitations were identified in this study. Firstly, it utilised a web-based survey shared through email and social networks, which may have been affected by self-selection bias. Secondly, the ability to make causal inferences is limited due to its cross-sectional design, so a longitudinal design which examines the long-term effects of the pandemic on lecturers is suggested. Finally, in the multiple model with student-related burnout as the outcome, the normality of residuals was not observed and thus the results of this model should be interpreted with caution. Despite such limitations, this study has several points of strength. Although in recent years, the study of burnout has been the subject of international research, studies in Portugal are still scarce (Mota et al., 2021). Therefore, this study contributes to increase the knowledge about the phenomenon in this professional class. The use of the CBI scale is also a strength, to the extent that it allows assessing different domains of life that may contribute to teachers’ professional fatigue and burnout. In addition, it allowed identifying the different contributions of socio-demographic and psychological variables on the three burnout dimensions. Finally, it was possible to identify that psychological resilience and life satisfaction function as protective variables against burnout, which leads us to make proposals for health programmes with a view to enhancing these qualities in teachers.

Future research is need. Since burnout is linked to job demands (e.g., quantitative, qualitative, and organizational demands) and job resources (e.g., feedback, rewards, job control, participation, job security, and supervisor support, perceptions of fairness) (Madigan and Kim, 2021; Naidoo-Chetty and du Plessis, 2021) future works should explore what sociodemographic and phycological variables affect different dimensions of burnout. In this study, psychological resilience and life satisfaction appear to be protective factors against burnout, it is recommended that future research explore the mediating roles of resilience and life satisfaction in the relationship between stress, depression and burnout amongst lecturers.

## 5 Conclusion

The results of this study indicate that high levels of personal burnout are experienced by 50% of lecturers, 37% experienced work-related burnout and 11% experienced student-related burnout. Each of the three burnout dimensions was linked with a particular set of covariates, which corroborates the multidimensionality of the burnout syndrome. Psychological resilience and life satisfaction were shown to be protective variables against burnout. In this sense, higher education institutions need to develop and provide health programs which promote and sustain resilience in lecturers. By observing the link between each dimension of burnout and specific sets of covariates, it is possible to identify risk factors that may predispose lecturers to burnout. This understanding is essential for developing effective intervention strategies. It is important to support teachers in developing skills to face unexpected challenges and maintain their mental health in situations of prolonged stress. Educational institutions and managers play a fundamental role in creating working environments that promote the resilience and mental health of teachers. Higher education institutions must recognize the impact of the work environment and organizational culture on faculty mental health and take proactive measures to improve this environment. These institutions can implement support strategies such as educational technology training, professional development programmes, emotional support resources, and workload flexibility. It is essential to consider teachers’ concerns to ensure they can provide quality education to students, even in emergency remote learning situations.

On the other hand, psychoeducational programmes like mindfulness-based interventions may improve these protective factors, and safeguard teachers against occupational stress and burnout.

## Data availability statement

The raw data supporting the conclusions of this article will be made available by the authors, without undue reservation.

## Ethics statement

The studies involving humans were approved by Ethics Committee of the São João Hospital Center (98/2020, on 29 May, 2020). The studies were conducted in accordance with the local legislation and institutional requirements. The ethics committee/institutional review board waived the requirement of written informed consent for participation from the participants or the participants' legal guardians/next of kin because all participants gave informed consent online in compliance with guidelines for clinical research.

## Author contributions

LC: Conceptualization, Data curation, Formal analysis, Investigation, Methodology, Writing – original draft, Writing – review & editing. CS: Conceptualization, Data curation, Investigation, Methodology, Writing – original draft, Writing – review & editing. AR: Data curation, Investigation, Writing – original draft, Writing – review & editing. SM: Data curation, Investigation, Writing – review & editing. JS: Data curation, Investigation, Writing – review & editing. TA-L: Data curation, Investigation, Writing – review & editing. CM: Data curation, Investigation, Writing – review & editing. AT: Conceptualization, Data curation, Formal analysis, Investigation, Methodology, Writing – review & editing. ID: Conceptualization, Data curation, Investigation, Writing – review & editing.
